# Parenting When Children Have Lyme Disease: Fear, Frustration, Advocacy

**DOI:** 10.3390/healthcare7030095

**Published:** 2019-08-08

**Authors:** Emilie M. Gaudet, Odette N. Gould, Vett Lloyd

**Affiliations:** 1 Department of Psychology, Mount Allison University, Sackville, NB E4L 1C7 Canada; 2 Department of Biology, Mount Allison University, Sackville, NB E4L 1G7 Canada

**Keywords:** Lyme disease, children and Lyme disease, Lyme families, children with complex medical conditions, parents as advocates, Lyme disease in Canada

## Abstract

Increasing numbers of Canadians, including children and adolescents, are being infected with *Borrelia burgdorferi* and contracting Lyme disease. In the present study, we provided a qualitative analysis of written correspondence produced by 23 parents of children and adolescents with Lyme disease. The goal of this study was to investigate how medical and psychological issues were highlighted by parents describing their family’s Lyme disease experiences. The results suggest a series of four stages in these families where satisfactory treatment had not been obtained over months or years. The experiences of parents evolved from feelings of worry for the child to frustration with the lack of a helpful treatment, to mistrust of physicians’ actions, and, in some case, to a rejection of the conventional health care system as a whole. Improved diagnostic testing and treatment guidelines, as well as family-centered practices of medical care were proposed as important features for improving the experiences of families living with Lyme disease.

## 1. Introduction

Canada is providing an increasingly viable habitat for ticks [1]. The path of migratory birds, the resurgence of deer populations, changes in land use, and most importantly climate change have all been shown to contribute to the expansion of habitats for the tick species that carries Lyme disease [2,3,4]. This expansion of tick populations is expected to increase the public health risk of Lyme disease within Canada. [1,5,6]. Indeed, it has been reported that the percentage of people in Eastern Canada at risk of contracting Lyme disease will increase from 18% in 2010 to 82% in 2020 [5]. Even surveillance data, which is believed to greatly underestimate the incidence of Lyme disease [7], showed a six-fold increase in the number of reported cases from 2009 to 2015 [8]. Of particular interest in the present context is the frequent finding that children and adolescents often contract Lyme disease [9]. For example, a Canadian national study revealed a bimodal distribution with the most cases appearing in individuals aged 5 to 9 and 45 to 74 years [8] and a study of clinical cases of Lyme disease in central Canada (Ontario) found that the age groups most commonly hospitalized for Lyme disease were 15 to 19-year-olds and 40 to 44-year-olds [10], results consistent with international findings [8,10].

Diagnosing Lyme disease has been shown to be a complex and contentious issue, particularly when the bacteria have had time to disseminate throughout the body [2,4,11]. At the acute phase, if the physician can document the erythema migrans rash and exposure to an endemic site, a probable diagnosis can be made by the physician, and antibiotics prescribed [12,13]. However, some studies have suggested that many individuals infected with Lyme disease do not experience the erythema migrans rash, rendering such diagnoses more challenging [8,14]. In later stages, (i.e., disseminated Lyme disease) or when the exposure to an area where infected ticks are endemic is not established, laboratory tests are required [7,15], and the sensitivity and effectiveness of these tests continues to be debated [2,4,11]. As the dissemination progresses, varied symptoms are experienced, including neurologic problems such as facial palsy, cardiac symptoms, chronic pain, and arthritis [2,7,8]. This diagnostic difficulty can be compounded by the lack of knowledge concerning Lyme disease on the part of health-care practitioners in emergent endemic areas [16,17]. Due to the difficulties surrounding diagnosis, many Canadians report feeling pushed out of the mainstream Canadian medical system to seek supportive and effective care from either international physicians or alternative health care providers [18]. 

Qualitative studies [19,20,21] exploring the experiences of patients in the United States as they navigate the healthcare system have emphasized the difficulties of seeking a diagnosis for symptoms of disseminated Lyme disease. Important themes highlighted in one set of interviews [10] included (a) feelings of frustration, (b) a long road to diagnosis, (c) financial stress, (d) self-advocacy, (e) validation once a diagnosis is found, and (f) hopefulness. In a similar study [20], four themes emerged: (a) a change in health as well as the social impact of the disease, (b) doubts about the future, (c) strained doctor-patient relationships, and (d) the use of unconventional therapy to treat the disease.

Although children are often affected by Lyme disease [8,10], the experience of families in which children suffer from the disease has received very little research attention. However, some related work has addressed the impact of other chronic diseases in childhood (for reviews see [22,23,24]). Caring for children with chronic pain, for example, has been found to be stressful for parents and often involved a change in how the parental role was defined [25]. Parents reported having to put their lives on hold to care for their children, and a sense of hopelessness and failure was reported by the parent when the child’s “normal” life changed [25]. Being the parent of a chronically ill child led to financial stress due to the cost of specialised healthcare for the child and the need to have low-income jobs to get flexible work hours in order to provide care to the ill child [26]. When a sample of caregiver parents to chronically ill children was compared to a sample of parents of healthy children, it was found that the mothers who were caregivers to their children reported working less and spending less time on leisure activities [27]. Stressors were particularly acute for families living in rural areas due to health facilities with fewer staff, a smaller selection of health providers, and fewer pediatric facilities [28].

In conclusion, Canadian families are at an increasing risk of having one or more members with Lyme disease, yet the diagnosis and treatment of the condition can be lengthy and complex. It is also clear that when children are affected by complex medical conditions, the parents, as well as the children, experience considerable distress. In the present study, we used a qualitative approach to investigate the experiences of parents whose children have Lyme disease. The purpose of this qualitative approach was to better understand the reality of families affected by Lyme disease. By asking participants to write a letter about their experiences as parents whose children have Lyme disease, we provided them with the opportunity to describe their experiences and concerns and their perception of how they had been treated by the healthcare system while trying to find treatment for their child.

## 2. Materials and Methods

### 2.1. Participants

Participants were recruited through two Canadian Lyme disease support groups. The first phase of recruitment involved contacting parents who had previously participated in a call to action led by a support group by writing a letter about their family’s experience with Lyme disease. The support group organizers contacted the parents and, with their permission, provided their contact information to the researchers, who then invited them to participate in this study. The participants were offered the option to submit their original letter or to send an updated version. Fourteen participants were recruited using this approach. Nine additional participants answered online social media posts from this support group and from the Canadian Lyme Disease Foundation. The posts consisted of a short recruiting message from the research team describing the study and inviting parents to contact the researchers by email or telephone if they wished to participate. Potential participants who contacted the researchers were provided with more information about the study. The Lyme disease support groups did not select specific participants and did not have input into study design, analysis, or outcome. In total, 23 parents from 5 different provinces participated in the study and acknowledged informed consent via email. All the participants who self-identified as having a child with Lyme disease were included, whether or not serological evidence of Lyme infection was present. 

### 2.2. Materials and Procedure

When participants contacted the primary researcher via email, they were informed of the nature and purpose of the study and invited to provide answers to a list of demographic questions. Participants were asked to provide written consent in their reply email and to send the researcher a letter describing their child’s or their family’s experience with Lyme disease. This methodology was approved by the Mount Allison University Research Ethics board (approval number #102216).

The letters were analyzed using a thematic analysis [29]. Preliminary codes were generated by two researchers after independently reviewing letters. The two researchers then went through letters one by one to create a complete list of codes until saturation was achieved, as indicated by the fact that no new codes were being identified [30]. Next, the two researchers discussed the inter-relationships between the codes, and through careful deliberation, guided by the research question, organized the codes into themes. Finally, the first author reread all the letters to (a) confirm the preliminary codes, (b) confirm thematic saturation, and (c) identify quotes that best represented each of the themes. It was previously established that qualitative methodologies are valuable in healthcare research for providing insight into patients’ and healthcare providers’ experiences that may be helpful in improving healthcare practices [31].

## 3. Results

Demographic information for the 23 parents that participated in the study is provided in Table 1.

When asked to produce a written statement describing their experience with their child’s Lyme disease, most participants produced documents that upon analysis revealed a relatively clear set of four stages that began with the onset of illness (a) Diverse Symptoms, (b) Tests and Specialists, (c) Dismissal, and (d) Seeking Care Outside the Conventional Health Care System. The twin themes of the parents’ (a) emotional reactions and (b) perceptions of the physician’s role were highlighted at each stage. De-identified verbatim quotes from the letters are provided in italics.

**Stage 1: Diverse Symptoms**: for many families, the Lyme disease experience began when the child displayed a variety of symptoms that parents subsequently identified as being consistent with Lyme disease. Particularly upsetting for the parents was the sudden and seemingly inexorable decline of a previously active and healthy child ((*I watched) my child go from healthy to wheelchair in a matter of weeks*) and the psychological distress experienced by the ailing child (*he continued to get worse, (h)e was crying all day, (s)ad about being unable to play with his brother, sad from the pain in his head).* As the symptoms continued over weeks and months, parents also worried that their child was missing important milestones (*she was not able to undertake the many extra-curricular activities that often define the teenage years (…) she has missed out on a lot and will never be able to get those years back (…) she is being left behind).*

The onset of a very wide range of symptoms was difficult to deal with and adapt to—both for the child and for the parent. The complete list of symptoms described in the letters is presented in Table 2. Although frustrated with the physicians consulted, some parents also acknowledged that the diverse and evolving set of symptoms was challenging for physicians (*his symptom list was extremely large and unconnected to each other which didn’t help with getting a diagnosis).* At this early stage, many parents were at a loss for an explanation of their child’s condition, but many others described a bull’s eye rash to the physician, and suggested Lyme disease as an explanation that was rejected (*my youngest child, who was 10 at the time, has TWO BULLS EYE RASHES on her body (…) so I took my child to that same family doctor and (the doctor said)“it can’t be Lyme. There’s no Lyme in BC”).* For the most part, parents sought help from physicians, and physicians proceeded to a series of tests and referrals in an attempt to find a diagnosis to explain the reported symptoms.

**Stage 2: Tests and Specialists**—One of the most consistent themes across the participants’ descriptions of their experiences was that the children were subjected to many tests, referred to many specialists, received many treatments and were given many diagnoses the parents considered incorrect. Table 3 presents full lists of the different diagnostic tests, specialists, and trial treatments reported by the study participants. As one parent reported, “(*My child) has no diagnosis in Canada despite seeing over 22 Canadian specialists between 2007 (…) until 2015*”. The distress of the repeated negative results was particularly acute. As one mother stated, “*I think this was one of the worst times for me because I found myself hoping, actually praying that something would show up (on the tests), that they would see something wrong so they had no choice but to address it. It made me feel like a terrible mother, who wishes for something to show up on a scan of their child?*” 

With the child undergoing such an intensive set of medical procedures, the negative impact was felt by the whole family (*our family will be forever changed because of Lyme*). Many parents highlighted the financial costs incurred from reduced work hours and travel to seek care. One mother, a professional who had had to leave her job to care for her ill daughter, summarized the financial costs (*I would estimate my lost earnings to be in the order of $30,000–$40,000. Add to this the extraordinary costs incurred over the years in an effort to find out what was wrong and to address the many symptoms (…) to be in the order of $20,000–$25,000).*

For some parents, the perception that specialists worked in silos and did not acknowledge the full array of symptoms experienced was particularly frustrating (*not one specialist or doctor considered Lyme disease or even made an effort to consider other clinical symptoms that happened to be outside of their own specialty).* As parents further investigated Lyme disease, they also became aware of the controversies surrounding Lyme disease testing and were more frustrated with physicians’ unwillingness to consider a Lyme disease diagnosis. In many cases, physicians refused to test for Lyme disease, but in others, a test was carried out but the possibility that the negative results were false was rejected. One mother concluded “*if only doctors were trained to recognize and treat her symptoms instead of relying on a blood test that is known to be inadequate at best those costs and invasive tests could have been avoided.”* Table 4 presents the broad array of diagnoses that the children in this study were assigned.

Finally, for many parents, this was also the stage of their child’s illness where they found themselves becoming an advocate who had to fight the health care system to ensure their child’s well-being (*I read about Lyme every single day. I have tried to take breaks from this, just for my own mental health and peace of mind, but have not lasted very long because this is my daughter and I would do anything for her to be well again*).

**Stage 3: Dismissal**: As part of the Lyme experience, many parents report reaching a point where the doctor would dismiss their case or give them a mental health-related diagnosis (*she said this was all vague and that he was making it all up for attention. (…) she did not feel she needed to assess him because this was not real).* Another parent wrote, “*as a child of 15 in 2008 she was dismissed from countless medical appts with doctors stating, just ‘take some Tylenol and get on with your life” or ‘lab work is normal, there is nothing else I can do’ (…) she felt put down, demoralized, and abandoned by the mainstream medical community”.* Still another parent wrote, “*they are not going to further investigate why my child can’t walk. All my child needs, in their collective “professional” opinion is exercise and a psychiatrist”.*

Some parents felt not only the stigma of a mental health diagnosis, but also felt afraid that their advocating for further treatment for their child would lead physicians to label them as abusive parents. Some parents were accused of enabling their child’s malingering *(but even after we had a positive Western Blot from an American lab we were told, here in Canada, that “labs for profit” cannot be trusted (…) that all my child needed to get well was to spend more time with their friends and that my husband and I had to stop enabling their behavior).* Other parents were accused of creating the problem as part of their own mental health issues (*every doctor visit is like walking into a field of land mines. We never know if we are going to get reported for Munchausen by proxy and have our kids taken away from us. This is a very real threat and a terrifying thing to face when all we want is for our kids to be well).*

Parents reported believing that at least some of their experiences are unique to Lyme disease families (*the sad part is most people with a chronic and debilitating illness only have to fight to regain their health—they do not have to fight their medical system to get necessary testing, support, and resources*). Indeed, as parents became more frustrated with their physician’s dismissal of their child’s health concerns, many also became increasingly informed about Lyme disease and convinced that a diagnosis of Lyme disease was warranted. This, in turn, created more problems (*I began to dread going to the ER or to the doctors, as once the word Lyme enters your dialogue, the abuse, ridicule, anger, dismissal, eye rolling, and ignorance begins, further traumatizing you when you are in a weakened, vulnerable and frightened state. People with chronic Lyme disease are marginalized, abandoned, ignored, lectured, yelled at, belittled, neglected, and dismissed*).

**Stage 4: Seeking Care Outside the Conventional Canadian Health Care System***:* At the time of writing the letter analyzed in this study, some parents were still seeking care within the mainstream health care system, although none were very hopeful of success (*we are trying our best but feel like we are treading water at best. We are not able to make progress and feel like the medical system is against us).* Many others were in the process of obtaining care outside of the Canadian mainstream health care system often through support from other people suffering from Lyme disease (*I often shudder at the thought of where he would be had we not essentially been “diagnosed over the backyard fence” type idea).* In some provinces, naturopathic doctors are able to prescribe antibiotics, and for many parents, finding a naturopathic doctor who was knowledgeable about Lyme disease and willing to be an ally was described as an intensely positive experience (*our Lyme (naturopathic) doctor has been a saving grace. Before we met him, my son was an empty shell of a child (…) he gave him back his childhood and I am forever grateful to doctors like him that actually listen to their patients and find answers)*.

For many parents, their experiences within the Canadian health care system while seeking care for Lyme disease for their children was an intensively negative experience that led them to lose trust in mainstream doctors. For some participants, this meant that physicians would protect each other rather than care for patients (*the experience of others is a clear warning: if you complain about a doctor’s treatment around the issue of Lyme disease, you get ‘blacklisted’ among the medical profession and doctors then will refuse to take you on as a patient)*. Other parents held the belief that some physicians wanted to help their patients but were hampered in doing so by medical authorities (*I am absolutely devastated to learn that our pediatrician has been ‘contacted’ and ‘warned’ and that she can no longer help my daughter for fear of losing her license).* A similar statement was made by another parent “*I can’t say that I blame him as I would not want to lose my license to practice in that way either, but the system is very sick and flawed when you don’t truly allow your bright minds to use them but instead control them and keep them in a box”.*

Parents also commented that when they found a health care provider who supported the diagnosis of Lyme disease, other health care providers often reacted with a certain amount of derision or hostility. One parent wrote (*I tried to avoid mentioning Lyme disease as I quickly learned that it would result in a barrage of derogatory comments or condescending statements about us and the ‘zealots’ who would dare treat us “just for money”, or how the labs in the US just making everything positive just to get more money*).

At this point in their journey, many parents felt a deep sense of betrayal with the medical system (*to be honest, by this time, I had completely given up on the western medical system and had lost faith in them*).

For some parents, this betrayal came not only from the health care system, but, by extension, the country as a whole (*forcing the sick to leave their country to receive the treatment that they desperately need to regain a decent quality of life, is sadly something that Canada will look back at one day and hang their heads in disbelief and shame!*)

Many parents ended their letters by stating that one important consequence of their experience had been becoming strong advocates for their children’s care, but also for the care of all people affected by Lyme disease. Indeed, this was the motivation that led many to take time away from a routine that was already overwhelming in order to participate in the study (*as horrible as this has been, I firmly believe that everything happens for a reason and that my job now is to spread awareness about this disease because it can happen to anyone. If I can help even one more person, then it won’t all be in vain).*

**Figure 1:**
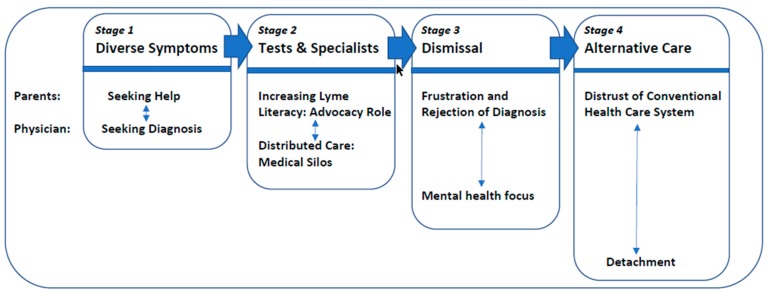
Parents’ perception of children’s Lyme disease care experiences.

In conclusion, the analysis of the letters written by parents of children suffering from Lyme disease suggests a series of four stages, each incorporating the experiences of the parents with their perception of the health care system. The stages are represented in Figure 1.

These experiences reveal successive stages in which parents feel considerable emotional distress, which evolves from feelings of worry for their child to frustration with the lack of a helpful treatment, to a mistrust in the physicians’ actions to, finally, a rejection of the health care system as a whole. When the medical care experiences began, physicians were seen as active partners seeking a diagnosis for the symptoms observed. However, as time progressed, they were perceived as focusing more and more on laboratory tests related to isolated symptoms rather than on a patient-centered approach. Some physicians eventually proposed psychological or psychiatric causes for the situation, or at the very least were perceived as giving up the search for a medical cause for the reported symptoms. Thus, although the physician and parent were working together at the beginning of the process, with both trying to find an explanation for a series of confusing symptoms, by the end of the process, the two were often estranged and seemingly frustrated with each other’s actions.

## 4. Discussion

The purpose of the present study was to gain an understanding of the experiences of families affected by Lyme disease who did not receive prompt diagnosis and treatment. To a great extent, our findings reinforce those obtained in previous studies of people living with Lyme disease [18,19,20,21] and of parents with children with chronic illnesses [22,23,24,25,26,27,28]. Particularly striking here were the experiences of the child’s (or adolescent’s) symptoms being dismissed as being psychological in nature and possibly due to bad parenting. Similarly, parents of children with attention deficit hyperactivity disorder (ADHD) have reported that teachers and other professionals referred to the child’s condition as being due to deficient parenting rather than to a medical cause [32].

Underlying much of the negative experiences reported by these parents was lack of knowledge about the disease within the medical community—presumably due to the relatively recent increase in the frequency of Lyme disease infections in Canada. In two studies carried out in Quebec in 2015 and 2017, [16,17] medical practitioners agreed that they needed more education on Lyme disease and many reported that they had requested laboratory tests when they were perhaps not needed. The diversity of clinical manifestations, the problems with two-tiered testing, and the possibility of chronic Lyme disease when no treatment or limited treatment was provided render appropriate diagnosis particularly challenging [33]. The lack of prompt and accurate diagnosis results not only in emotional and financial difficulties for the families affected, but also in increases in healthcare costs due to the large number of tests and procedures needed when a diagnosis [33] of Lyme disease is not considered in the differential diagnosis process. A further concern, of course, is that parents who give up on conventional medicine could, in some cases, jeopardize their child’s wellbeing by attempting non-validated treatments. Care outside of the “mainstream Canadian health care system” is a very broad category that includes both American physicians and naturopathic doctors licensed to prescribe antibiotics, as well as alternative and faith-based treatments.

Recent initiatives by the Canadian government may improve the situation. A report published in May 2017 entitled, “Lyme Disease in Canada: A Federal Framework” [34] outlines measures to be taken by the government to improve surveillance, education and awareness surrounding Lyme disease. The report also proposes guidelines and the best practices for treatment. Although it can be expected to take time for best practices to be identified and achieved across the country, it should be noted that the letters described in the present study were written a year after the report was published and these parents/advocates did not describe any improvement in how their local health care practitioners dealt with Lyme disease. Of course, one could argue that the failure of the Canadian health care system to treat children for Lyme disease is entirely appropriate if the children did not in fact have Lyme disease. Indeed, many of the cases described included family members who did not have unambiguous Canadian serological evidence of infection. However, regardless of whether the children had Lyme disease, a related Lyme-like disease or some other condition, the impact of poor health on the children and on their families was real and the suffering considerable. It is also of note that some of the families noted improved health when the children were treated for Lyme disease.

One issue highlighted in our study was the need for more research on the human-to-human transmission of Lyme disease. Some families in our sample had parents and multiple children with Lyme disease, often from an early age, warranting the question of whether *Borrelia* bacteria can only be transmitted environmentally or whether there can also be trans-placental transmission resulting in congenital infections. Although a recent study addresses this question [35], much remains to be learned about not only the frequency and conditions for such transmission, but also which of the many species of *Borrelia* might be most commonly transmitted in utero as well as the effect on the child’s health.

Clearly, this study has many limitations. A first, and very important issue is that we only had access to one version of events and could not obtain a detailed account of doctors’ and medical professionals’ experiences while treating these Lyme disease families. While accounts from past qualitative research studies and the participants of this study were quite consistent [18,19,20,21], suggesting generalizable experiences in the Lyme disease community, further studies including healthcare professionals’ perspectives would shed light on the difficulties of treating Lyme disease. A second limitation this study faces is that the sample of participants could not be generalizable to the entire Lyme disease community: our sample was limited to families who suffered many years from the disease and its direct and indirect impacts on family life. Thus, this study did not include participants who received a prompt and accurate diagnosis of Lyme disease and subsequently received appropriate treatment. It would be helpful in future research to identify the characteristics of patients and clinicians where positive experiences with Lyme disease diagnosis and care were present. Finally, it should also be noted that most participants in the present study were women, and the extent to which gender played a role in the experiences described deserves attention.

## 5. Conclusions

In conclusion, improved diagnostic testing and treatment guidelines are clearly and urgently needed for all Lyme disease patients. Moreover, for at least some families, the present standards of medical care surrounding Lyme disease are insufficient and more effective approaches are needed. The use of a health navigator or a team-based approach for dealing with children who have complex and chronic health conditions is being attempted in some jurisdictions and may be particularly helpful for children suffering from a multitude of symptoms linked to Lyme disease [36,37]. Another solution that could help reduce tensions between medical professionals and parents in Lyme disease families would be the implementation of patient-centered or family-centered practices of care. Family-centered care aims to empower patients to search for care and allows for healthcare professionals to better understand the entire family’s circumstances and situation [38]. This approach helps ensure that families’ needs are understood and met and fosters respectful relationships between parents and healthcare staff—something that is often lacking for Lyme disease patients at the present time.

## Figures and Tables

**Table 1:** Sample characteristics.

Demographic Information ^1^	Number of Participants (*n* = 23)	Percentage of Participants
Gender of letter writer		
Female	20	87%
Male	0	0%
No answer provided	3	13%
Number of parents in household		
One	3	13%
Two	18	78%
No answer provided	2	9%
Number of children in family		
One	3	13%
Two	10	43%
Three	4	17%
Four	4	17%
No answer provided	2	9%
Children in family with confirmed or suspected Lyme disease		
One	13	56%
Two	5	22%
Three	3	13%
No answer provided	2	9%
Province		
Ontario	11	48%
Alberta	4	17%
British Columbia	3	13%
Nova Scotia	1	4%
Quebec	1	4%
No answer provided	3	13%
Area		
Rural	6	26%
Urban	12	52%
No answer provided	5	22%

^1^ Some participants did not answer all demographic questions.

**Table 2:** List of reported early symptoms.

Type of Symptom	Symptoms Reported
Pain	Arthritis/generalized pain/spinal pain
	Migrating joint pain/swollen and inflamed joints/twisted fingers
	Headaches/pressure/sudden pain/brain feels inflamed
	Pain in limbs/stabbing pain/restless legs
	Pain in stomach/chest/ribs/stomach
Developmental Issues	Delayed growth/delayed puberty
	Poor in-utero growth
Psychological Symptoms	Attention deficit/hyperactivity
	Anger/Aggression/Irritability
	Altered personality/mood swings
	Anxiety/panic attacks
	Depression
	Hallucinations/paranoia /psychosis
	Nightmares
	Obsessive compulsive disorder
	Tics (blinking/throat clearing/toe walking/vocal tics, movements)
	Self-harm
	Social phobia/withdrawal
	Suicidal thoughts
Neurological & Cognitive Symptoms	Brain fog/confusion/memory loss
	Cognitive losses or delays (reading/speech/math/writing)
	Inflamed cranial nerves
	Facial droop/palsy
	Fainting
	Sensory issues (vision, hearing, taste, smell)/nystagmus
	Tingling/numbness/tightness in extremities
	Tremors/seizures
	Uncontrollable muscle contractions
	Vertigo/dizziness/poor balance
Various Physical Symptoms	Anal fissures/rectal bleeding
	Appetite loss/anorexia
	Baker’s cysts
	Bed wetting/frequent urination
	Blood and mucous in stool
	Breaking teeth/deformed fingernails
	Cardiac complications
	Croup (recurring)/relentless coughing
	Diarrhea/colicky/constipation
	Dry/red/blistering eyes/bags under eyes
	Ear infections
	Elevated C-reactive protein levels
	Elevated liver enzymes
	Fever
	Food/alcohol/heat intolerance
	Flushed cheeks/red lines on cheeks
	Gastric reflux/heartburn /drooling
	Herzheimer reaction
	High blood pressure
	Hormonal issues
	Insomnia/sleepwalking
	Irregular bloodwork
	Jaundice
	Low ferritin levels/low white blood cell count
	Mouth sores
	Nausea/vomiting
	Pneumonia
	Poor hair growth
	Rash:/hives/red, puple bumps/bullseye/ringworm/molluscum contagiosum
	Skin sensitivity/eczema /itchiness
	Short of breath at rest/shallow breathing /pale
	Sinus issues/colds/flu symptoms/phlegm
	Soaking sweats
	Swollen glands/thyroid/hypothalamus issues/lymph nodes
	Weakness/can’t stand or walk/bedridden/ exhaustion
	Weight gain/loss

**Table 3:** Use of the health care system.

Healthcare professionals & sites consulted ^a^	Allergist
	Clinical and metabolic genetics consultant
	Counselor/therapist
	Dermatologist
	Developmental pediatrician
	Ear, nose, and throat specialist
	Family doctor
	Gastroenterologist
	Hospital resident
	Immunologist
	Infection disease specialist
	Local emergency staff
	“Lyme literate” US doctor
	Lyme specialist
	Lymphatic massage drainage therapist
	Naturopath (Lyme literate and non)
	Neurologist
	Neurosurgeon
	Nurse practitioner
	Obstetrician/gynecologist
	Ophthalmologist
	Pediatric care teaching hospital
	Pediatric gastroenterologist
	Pharmacist
	Podiatrist
	Psychiatric ward/psychiatrist
	Rehabilitation hospital
	Rheumatologist
	Sleep clinic
	Social worker
	Urologist
Treatments prescribed and attempted	Acyclovir
	Adrenal fatigue pills
	Allergy medication
	Antibiotics (oral and IV) ^b^
	Anti-malaria drugs (Malarone)
	Anti-parasitic drugs
	Chinese acupuncture
	Chiropractic therapy
	Chronic complex disease program
	Cognitive behavioral therapy
	Cortisone
	Counselling
	CPAP machine
	Diet: sugar free, gluten free, yeast free, vegan, vegan keto, FODMAP
	Dietary supplements and vitamins (oral & IV) ^c^
	Essential oils/herbals/tinctures
	Exercise
	Eye patch
	Hydrocortisone cream
	Intravenous immunoglobin treatment
	Journaling
	Laser treatment
	Pain relievers ^d^
	Topical cream/ointment
	Psychiatric medications ^e^
	Physiotherapy
	PICC line
	Pool therapy
	Rife machine therapy
	Traditional Chinese medicine
	Tube feeding
Diagnostic tests conducted	Bloodwork ^f^
	Cardiac workup
	CT scan
	Electromyography
	Genetic testing
	Immunology tests
	Lumbar puncture
	Lyme screening test ^g^
	Mono tests
	MRI scan
	Muscle biopsy
	Nasal swabs
	Neurological testing
	Nerve conduction studies
	Endoscopies for throat/liver/pancreas
	Sleep-deprived EEG
	Specialized T-cell test
	Stool and urine sample tests
	Viral test
	X-ray

^a^ Some participants simply reported the number of professionals consulted, including 15 + specialists, 30 doctors, and 22 specialists. ^b^ Ceftriaxone, Clavulin, amoxycillin, doxycycline, minocycline. ^c^ Probiotics, B12 vitamins, prebiotics, gluthatione. ^d^ Ketamine, morphine, Dilaudid, Tramadol, Tylenol. ^e^ Fluoexetine, Ativan, Anti-anxiety medication. ^f^ Iron, potassium, autoimmune diseases, Lyme disease; ^g^ ELISA, Western blot, ELISPOT.

**Table 4:** Proposed diagnoses.

ADD/ADHD	Malnutrition
Anorexia	Mental disorder
Anxiety	Mosquito bite
Attention seeking	Multiple sclerosis
Celiac	Oversleeping
Chronic blepharitis/conjunctivitis	Pain amplification syndrome
Chronic constipation	PANDAS
Chronic fatigue syndrome	Patellofemoral syndrome
Conversion disorder	Pediatric migraines
Cryptosporidium	Polycystic ovary syndrome
Daycare syndrome	Post-concussion syndrome
Depression	Psychogenic causes
Double jointedness	Psychosomatic symptoms
Fybromyalgia	Stomach virus
Food sensitivities	Teething
Growth spurts/pains	Tourette’s syndrome
H1N1	Unexplained medical illness
Infectious mononucleosis	Virus
Irritable bowel syndrome	

## References

[1] Ebi K.L., Ogden N.H., Semenza J.C., Woodward A. (2017). Detecting and attributing health burdens to climate change. Environ. Health Perspect..

[2] Greig J.D., Young I., Harding S., Mascarenhas M., Waddell L.A. (2018). A scoping review of Lyme disease research relevant to public health. Can. Commun. Dis. Rep..

[3] Lieske D.J., Lloyd V.K. (2018). Combining public participatory surveillance and occupancy modelling to predict the distributional response of Ixodes scapularis to climate change. Ticks Tick Borne Dis..

[4] Githeko A.K., Lindsay S.W., Confalonieri U.E., Patz J.A. (2000). Climate change and vector-borne diseases: A regional analysis. Bull. World Health Organ..

[5] Leighton P.A., Koffi J.K., Pelcat Y., Lindsay R., Ogden N.H. (2012). Predicting the speed of tick invasion: An empirical model of range expansion for disease vector Ixodes scapularis. J. Appl. Ecol..

[6] Ogden N.H., Radojevic M., Wu X., Duvuuri V.R., Leighton P.A., Wu J. (2014). Estimated effects of projected climate change on the basic reproductive number of the Lyme disease vector Ixodes scapularis. Environ. Health Perspect..

[7] Lloyd V.K., Hawkins R.G. (2018). Under-Detection of Lyme Disease in Canada. Healthcare.

[8] Gasmi S., Ogden N.H., Lindsay L.R., Burns S., Fleming S., Badcock J., Hanan S., Gaulin C., Leblanc M.A., Russell C. (2017). Surveillance for Lyme disease in Canada: 2009–2015. Can. Commun. Dis. Rep..

[9] Centers for Disease Control and Prevention Lyme Disease Charts and Figures: Historical Data. https://www.cdc.gov/lyme/stats/graphs.html.

[10] Johnson K.O., Nelder M.P., Russell C., Li Y., Badiani T., Sander B., Sider D., Patel S.N. (2018). Clinical manifestations of reported Lyme disease cases in Ontario, Canada: 2005–2014. PLoS ONE.

[11] Borgermans L., Goderis G., Vandevoorde J., Devroey D. (2014). Relevance of chronic lyme disease to family medicine as a complex multidimensional chronic disease construct: A systematic review. Int. J. Fam. Med..

[12] Centers for Disease Control and Prevention (2018). Tickborne Diseases of the United States—A Reference Manual for Healthcare Providers.

[13] Government of Canada (2018). For Health Professionals: Lyme Disease. https://www.canada.ca/en/public-health/services/diseases/lyme-disease/health-professionals-lyme-disease.html.

[14] Glaude P.D., Huber A.M., Mailman T., Ramsey S., Lang B., Stringer E. (2015). Clinical characteristics, treatment, and outcome of children with Lyme arthritis in Nova Scotia. Paediatr. Child Health.

[15] Ogden N.H., Arsenault J., Hatchette T.F., Mechai S., Lindsay L.R. (2017). Antibody responses to Borrelia burgdorferi detected by western blot vary geographically in Canada. PLoS ONE.

[16] Ferrouillet C., Milord F., Lambert L., Vibien A., Ravel A. (2015). Lyme disease: Knowledge and practices of family practitioners in southern Quebec. Can. J. Infect. Dis. Med. Microbiol..

[17] Gasmi S., Ogden N.H., Leighton P.A., Adam-Poupart A., Milord F., Lindsay L.R., Barkati S., Thivierge K. (2017). Practices of Lyme disease diagnosis and treatment by general practitioners in Quebec, 2008–2015. BMC Fam. Pract..

[18] Boudreau C.R., Lloyd V.K., Gould O.N. (2017). Motivations and experiences of Canadians seeking treatment for Lyme disease outside of the conventional Canadian health-care system. J. Patient Exp..

[19] Drew D., Hewitt H. (2006). A qualitative approach to understanding patients’ diagnosis of Lyme disease. Pub. Health Nurs..

[20] Ali A., Vitulano L., Lee R., Weiss T.R., Colson E.R. (2014). Experiences of patients identifying with chronic Lyme disease in the healthcare system: A qualitative study. BMC Fam. Pract..

[21] Rebman A.W., Aucott J.N., Weinstein E.R., Bechtold K.T., Smith K.C., Leonard L. (2017). Living in Limbo: Contested narratives of patients with chronic symptoms following Lyme disease. Qual. Health Res..

[22] McCann D., Bull R., Winzenberg T. (2012). The daily patterns of time use for parents of children with complex needs: A systematic review. J. Child Health Care.

[23] Ward C., Glass N., Ford R. (2014). Care in the home for seriously ill children with complex needs: A narrative literature review. J. Child Health Care.

[24] Mattson G., Kuo D.Z., AAP Committee on psychosocial aspects of child and family health, AAP Council on children with disabilities (2019). Psychosocial Factors in children and youth with special health care needs and their families. Pediatrics.

[25] Maciver D., Jones D., Nicol M. (2010). Parents’ experiences of caring for a child with chronic pain. Qual. Health Res..

[26] George A., Vickers M., Wilkes L., Barton B. (2011). Financial implications for parents working fulltime and caring for a child with chronic illness. Aust. J. Early Child.

[27] Hatzmann J., Peek N., Heymans H., Maurice-Stam H., Grootenhuis M. (2014). Consequences of caring for a child with a chronic disease: Employment and leisure time of parents. J. Child Health Care.

[28] Murphy K.L., Kobayashi D., Golden S.L., Nageswaran S. (2012). Rural and nonrural differences in providing care for children with complex chronic conditions. Clin. Pediatr..

[29] Braun V., Clarke V. (2006). Using thematic analysis in psychology. Qual. Res. Psychol..

[30] Guest G., Bunce A., Johnson L. (2006). How many interviews are enough? An experiment with data saturation and variability. Field Methods.

[31] Neergaard M.A., Olesen F., Andersen R.S., Sondergaard J. (2009). Qualitative description—The poor cousin of health research?. BMC Med. Res. Methodol..

[32] Harborne A., Wolpert M., Clare L. (2004). Making sense of ADHD: A battle for understanding? Parents’ views of their children being diagnosed with ADHD. Clin. Child. Psychol. Psych..

[33] Stricker R.B., Fesler M.C. (2018). Chronic Lyme disease: A working case definition. Am. J. Infect. Dis..

[34] Government of Canada (2017). Lyme Disease in Canada: A Federal Framework. https://www.canada.ca/content/dam/phac-aspc/documents/services/publications/diseases-conditions/lyme-disease-canada-federal-framework/lyme-disease-canada-federal-framework-eng.pdf.

[35] Waddell L.A., Greig J., Lindsay L.R., Hinckley A.F., Ogden N.H. (2018). A systematic review on the impact of gestational Lyme disease in humans on the fetus and newborn. PLoS ONE.

[36] Charlton P., Azar R., Luke A., Doucet S., Montelpare W., Nagel D., Hyndman N., Thompson K. (2017). Falling through the cracks: Barriers to accessing services for children with complex health conditions and their families in New Brunswick. J. N. Brunswick Stud..

[37] Luke A., Doucet S., Azar R. (2018). Paediatric patient navigation models of care in Canada: An environmental scan. Paediatr. Child. Health.

[38] Dunst C.J., Trivette M. (1996). Empowerment, effective helpgiving practices and family-centered Care. Pediatr. Nurs..

